# A New Laser Ultrasonic Inspection Method for the Detection of Multiple Delamination Defects

**DOI:** 10.3390/ma14092424

**Published:** 2021-05-06

**Authors:** Tianfang Gao, Yishou Wang, Xinlin Qing

**Affiliations:** School of Aerospace Engineering, Xiamen University, Xiamen 361005, China; gaotianfanghit@163.com (T.G.); wangys@xmu.edu.cn (Y.W.)

**Keywords:** guided wave, laser, delamination, wavefield, local wavenumber, localized wave energy

## Abstract

Delamination is one of the most common types of defects for carbon fiber reinforced plastic (CFRP) composites. The application of laser techniques to detect delamination faces difficulties with ultrasonic wave excitation because of its low thermal conductivity. Much of the research that can be found in the literature has only focused on the detection of a single delamination. In this study, aluminum foil was pasted onto the surface of the composite so that it was vulnerable to ablation and could acquire a usable signal. Using a fully noncontact system with laser excitation at a fixed point and a scanning laser sensor, the effects of different aluminum foil sizes and shapes on the wavefield were studied for the composites; we decided to use a rectangle with 3 mm length and 5 mm width for laser excitation experiments. Wavefield characteristics of the composite plates were analyzed with single- and multi-layered Teflon inserts. Taking the time window for standard ultrasonic testing as a reference, the algorithms for localized wave energy with appropriate time windows are presented for the detection of single and multiple defects. The appropriate time window is meaningful for identifying each delamination defect. The algorithm performs well in delamination detection of the composites with one or multiple Teflon inserts.

## 1. Introduction

In the aircraft and wind turbine industries, carbon fiber reinforced plastic (CFRP) composites are widely used as structural components thanks to their light weight and high strength. Since CFRP structures are fabricated by bonding multiple layers of laminates with resin, one of the most common types of defects is delamination. Such defects can grow and affect the mechanical properties and structural integrity. Delamination often occurs between the composed laminates, so it is invisible from the outside; therefore, non-destructive testing (NDT) of composites is essential and challenging.

Ultrasonic testing is the most commonly used method for detecting defects in materials. Ultrasonic structural health monitoring (SHM) techniques [1,2,3] apply piezoelectric sensor arrays to detect composite damage; a small number of sensors can locate damaged regions. The piezoelectric transducer (PZT) is frequently utilized for excitation. However, if the piezoelectric sensor arrays are sparse, quantifying defects is difficult. Traditional ultrasonic NDT testing methods are mainly based on contact methods, and their efficiency is often low because they are mostly performed manually or semi-automatically. The necessity of a couplant limits the development of ultrasonic techniques [4]. With the advantages of noncontact detection and high spatial resolution, the laser scanning technique [5,6,7,8,9] has the potential to be applied for inspection of structural components, mainly by identifying wave pattern anomalies in the full wavefield caused by damage. The measured, guided wave has the advantages of sensitivity to a variety of structural defects and long propagation distance [10,11].

For the detection of defects in CFRP composites, the use of laser techniques for ultrasonic wave excitation faces difficulties because of it low thermal conductivity. Relevant studies have shown that a laser beam with a wavelength near 3.2 μm is the best for generation of ultrasonic waves in CFRPs [12,13], but such lasers are not widely used. Common lasers, e.g., Nd:YAG and CO2 lasers, have been used for ultrasonic testing. The laser Doppler vibrometer (LDV) is used for guided wavefield measurement because of its high sensitivity to out-of-plane displacement. To obtain usable laser ultrasonic signals without ablation in composite materials, the automated PZT/LDV or laser/PZT scanning system is used for delamination detection. In a PZT/LDV scanning system, the PZT excites the guided wave and the scanning LDV measures the guided wave; different transducers are chosen based on the requirements. Vibration patterns [14] were obtained by using a piezoelectric transducer at 80 V and frequencies ranging from 0 to 100 kHz. The curved signal acquired from the resonance phenomenon was applied to realize the detection of multi-component defects. In one study, a 20 kHz–1 MHz, 100 μs chirp excited by a broadband contact transducer was used for excitation and multi-frequency local wavenumber analysis to detect multi-ply delamination damage in CFRP composite specimens [15]. For laser/PZT scanning systems, a mirror scanner is often used to achieve laser scanning. In [16], the broadband piezoelectric transducer was used to receive signals and its cut-off frequency was 2 MHz, which was integrated by an omnidirectional amplifier. The imaging method of adjacent wave subtraction was developed for non-destructive evaluation of complex structures to highlight anomalous wave propagation. In [17], a system of scanning laser-generated ultrasound was used for the detection of 20 mm-impact delamination in a composite wing section with 2 mm thickness. The proposed local wavenumber estimation was mapped using a sliding wavenumber band-pass filter to maximize the wave energy of every grid point at a specific mode and frequency. Currently, complete noncontact laser ultrasonic systems are mostly used for metallic materials [11,18,19,20]. In this study, aluminum foil was glued to the surface of the composite so that it was vulnerable to ablation and could achieve complete noncontact laser ultrasonic testing.

There are many methods for delamination detection using the wavefield of a guided wave [21,22,23,24,25,26,27,28,29,30,31]. Damage indicators can be extracted from time domain [25], frequency domain [26], and wavenumber domain [27,28,29,30,31]. Wavenumber imaging methods have been studied extensively for the detection of a single delamination defect. A single delamination refers to one delamination through the thickness of the composite material. Frequency–wavenumber filtering [27] could remove the source waves and highlight the location of weak scatters related to delamination defects in a composite. A standing wave filter [28] was adopted for the identification and visualization of hidden delamination in composites. Filtering reconstruction imaging [29] confirmed that the increased wavenumbers were correlated with trapped waves of the delamination, and the energy map resulting from the wavenumber filtering was proven to be effective for single delamination detection. Spatial or local wavenumber [20,29,30] algorithms were applied to obtain wavenumber values at each spatial location by spatial or wavenumber windows; these algorithms highlighted the delamination damage with larger wavenumber values. The wavenumber adaptive image filtering method [31] was utilized to detect delamination at various impact energies. The Riesz transform was applied to the wavenumber index technique [32] for damage detection, which was a generalization of two-dimensional (2D) Hilbert transform. The robustness of this technology lied in the fact that it located the impact damages with high resolution near geometry/material discontinuities in the composite with stiffener. However, when delamination defect is created, it often occurs between multiple ply levels through the thickness of the composite. In [33], trapping energy increased on the surface of composite materials when multiple delamination defects existed through the composite thickness. In [34], the wavenumber analysis could identify the laminates between which multiple delamination defects occurred. In [15], multi-frequency local wavenumber analysis and a ply correlation technique were investigated for quantitative assessment of multi-ply delamination defects in CFRP composite specimens. The wave energy method or filtering reconstruction imaging is rarely studied for the detection of multiple delamination defects.

In this study, aluminum foil was attached to the surface of the composite, and the effects of different aluminum foil sizes and shapes on the wavefield were studied for the composites. Taking the time window for standard ultrasonic testing as a reference [35], the algorithms of localized wave energy with appropriate time windows are present for identification of hidden single and multiple delamination defects. A fully noncontact laser scanning system, using a pulse laser to excite ultrasonic waves and adopting a continuous laser to measure out-of-plane displacement, was established for delamination detection. Three specimens with delamination defects were used for verifying the algorithm.

This article is organized as follows. Section 2 presents the laboratory experimental setup, the effects of different aluminum foil sizes and shapes on the wavefield, and experimental data. Section 3 gives the wavenumber characteristics of composite with single and multiple Teflon inserts. Section 4 describes the algorithms of localized wave energy with appropriate time windows. Section 5 presents the experimental results. Section 6 concludes with a brief summary.

## 2. Experiment

### 2.1. Experimental Set Up

**Figure 1:**
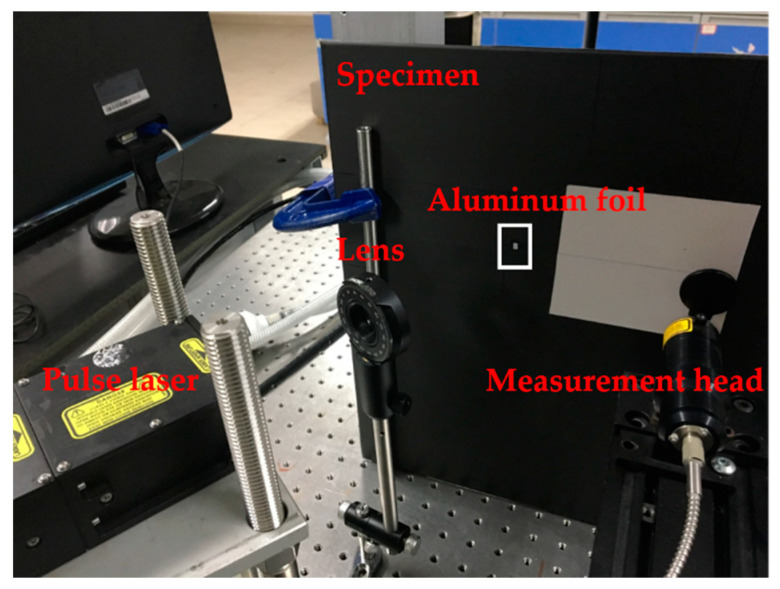
Experimental setup.

The scheme of fixed-point laser excitation and scanning laser sensor is one style of four excitation and sensing systems [18]. Such a scanning scheme can minimize the damage of pulse laser to specimens, although it reduces energy level and signal-noise ratio (SNR) of the generated ultrasonic waves. An overview of the experimental setup is shown in Figure 1.

**Figure 2:**
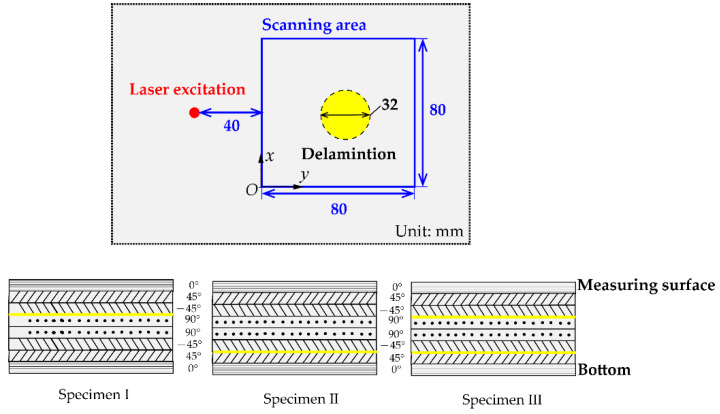
A schematic of the sensing layout and details of composite specimens containing the Teflon insert. There were three composite specimens with a stacking sequence of [0/±45/90] s. From the perspective of the through-thickness panel layup, a single Teflon insert was added to specimen I between the fifth and sixth layer, and to specimen II between the second and third layer; two Teflon inserts were added to specimen III, one between the second and third layer, the other between the fifth and sixth layer.

A Nd:YAG pulse laser is used to excite ultrasonic waves. The wavelength is 1064 nm, the pulse duration is 8 ns, and the maximum pulse energy is 55 mJ. Pulse laser was fixed on the optical platform and the energy (about 20 mJ) was set for excitation. The beam size needs to be adjusted by placing optical lens in front of the pulse laser. As the size of the laser beam becomes large, the amplitude of the direct waves and reflect waves increases when the peak energy density of the laser beam is constant [36]. In this study, the laser beam radius is set to 1 mm. A two-wave mixing (TWM) laser interferometer is applied for receiving ultrasonic waves. The signal is the out-of-plane displacement of the laser-excited ultrasonic wave, and it has been transformed into an electrical one represented by Volt. The sensing equipment includes a continuous laser with the wavelength of 1550 nm, an optical splitter and a TWM laser interferometer with sensitivity 3.9 × 10^−6^ nm(W/Hz)^1/2^. The system is manufactured by Intelligent Optical System Inc. (Torrance, NM, USA). The measurement head was installed on the X-Y scanning guide rail. The X and Y direction and origin of the scanning region are shown in Figure 2.

In the experiment, aluminum foil was attached on the excitation point to protect the surface from burning. The glue layer was made of acrylic acid with strong adhesion. The thickness of the bonded aluminum foil was 0.1 mm. Before the aluminum foil was pasted onto the composite, mark the excitation position. The aluminum foils with the 6 mm-diameter circle or the rectangle of 3 mm length and 5 mm width were studied for laser excitation. To get a high SNR, an average method of ten measurements was used at each sensing point. After the laser hits the aluminum foils several times in succession, it was also easy to cause ablation or breakdown. Therefore, the aluminum foil should be replaced regularly. In the experiment, the aluminum foil was replaced every two lines of laser scanning.

The specimens were the 1.5 mm crossply CFRP made of T300 prepreg materials with a stacking sequence of [0/±45/90] s, as shown in Figure 2. Since the material properties of CFRP are not known in advance, two specimens with single delamination defect at different layer positions were made to study the wavenumber characteristics related to the e material properties. Additionally, its purpose was also to analyze the wavenumber characteristics in the composite specimen with multiple delamination defects. Specimen I and specimen II were the composite plates with a single defect, and specimen III was the composite plate with multiple defects. From the perspective of the through-thickness panel layup, a single Teflon insert was added to specimen I between the fifth and sixth layer, and to specimen II between the second and third layer; two Teflon inserts were added in specimen III, one between second and third layer, the other between the fifth and sixth. The diameter of all delamination defects was 32 mm, and the distance between two Teflon center in specimen III was 10 mm. The TWM laser interferometer scans an area of 80 mm × 80 mm with a spatial resolution of 1 mm.

### 2.2. Experimental Data

**Figure 3:**
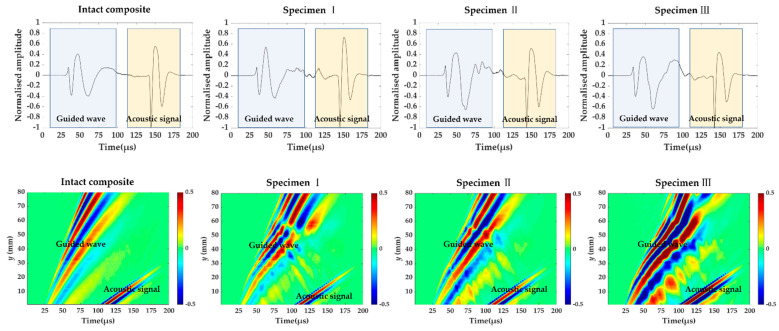
The signals measured at (40 mm, 55 mm) and time–space wavefield at *x* = 40 mm in the intact composite plate and three specimens.

Figure 3 shows the signals measured at (40 mm, 55 mm) and time–space wavefield at *x* = 40 mm in the intact composite plate and three specimens. The total signal includes guided wave and acoustic signal. The acoustic wave here refers to the signal propagated through the air. The measurement time of the acoustic signal coincides with the time taken for the wave to travel a distance of a certain distance at a speed of 340 m/s in the air. In addition to the acoustical signal, the prominent component of guided wave is the A_0_ mode because it is the main component of the out-of-plane displacement. The guided wave velocities in the intact composite plate are different from one in three damaged composite plates. Additionally, the velocity of the guided wave becomes slower for specimen I, II and III, because the slope of the line is smaller compared to one in the intact composite.

### 2.3. The Effect of Aluminum Foil Size and Shape on the Wavefield

**Figure 4:**
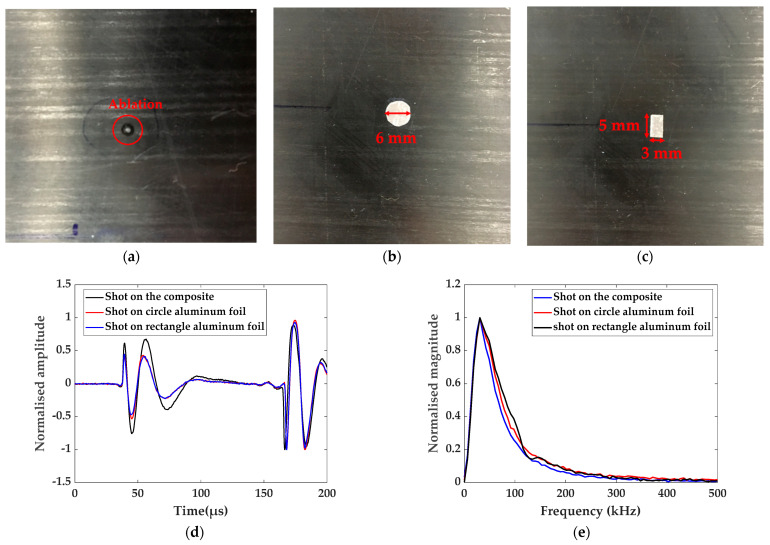
(**a**) Ablation caused by 20 consecutive laser radiation on the composite, (**b**) circular aluminum foil with 6 mm diameter, (**c**) rectangular aluminum foil with 3 mm length and 5 mm width, and (**d**) the signal of laser shot on composite, circular aluminum foil and rectangular aluminum foil, (**e**) corresponding signals in the frequency domain

When the surface of a solid is illuminated by the laser beam, the thermal energy of the electromagnetic radiation is absorbed by the region and local temperature is produced. Then, the physical process of thermal expansion occurs and it produces stresses and strains in the solid. The sudden stresses in the illuminated region excite a transient displacement field. For the composite, the thermal conductivity is low and it is vulnerable to ablation. Figure 4a shows the ablation caused by 20 consecutive laser radiation on the composite. Additionally, ultrasonic wave acquired by the laser with low power intensity has the low SNR. Therefore, it is necessary to protect the surface of the composite from ablation while ensuring the high SNR of the guided wave.

The aluminum foil with thickness 0.1 mm was pasted onto the surface of the composite to obtain the wavefield. In order to eliminate the influence of aluminum foil size and shape on the wavefield, the circular aluminum foil with 6 mm diameter and the rectangular one with 3 mm length and 5 mm width were prepared in the experiment, as shown in Figure 4b,c. Figure 4d shows the signals of laser shot on the composite, circular and rectangular aluminum foil with the same power intensity and average times. The arrival time of the ultrasonic wave peak and valleys acquired by laser shot on aluminum foil is basically the same as one by laser shot on the composite. Figure 4e gives corresponding signals in the frequency domain. There is no difference in terms of the signals of laser shot on aluminum foil in the frequency domain. The frequency band of the signal obtained by laser shot on aluminum foil is wider, which may be related to the properties of the materials. For the three signals, the position of main frequency is the same. It can be concluded that the aluminum foil can be used for delamination detection and the size and shape of aluminum foil have no effect on the signal.

**Figure 5:**
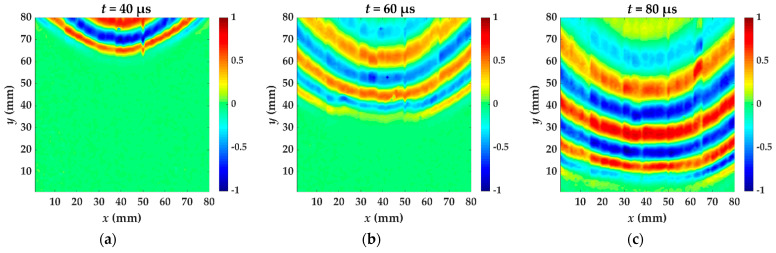

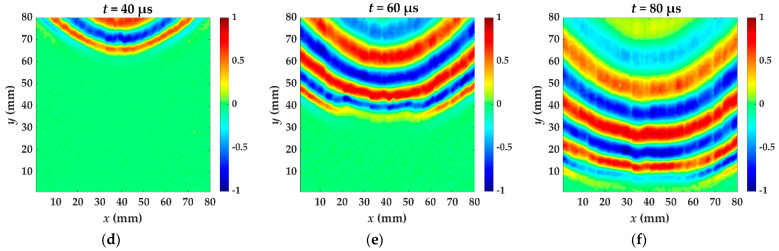
The wavefield snapshots (**a**–**c**) are at 40, 60 and 80 μs acquired by laser shot on circular aluminum foil. The wavefield snapshots (**d**–**f**) are at 40, 60 and 80 μs acquired by laser shot on rectangular aluminum foil.

The aluminum foil was replaced every two lines of laser scanning and the acquired wavefield snapshots at 40, 60 and 80 μs by laser shot on two aluminum foils are shown in Figure 5. The wavefront shapes of two wavefields are almost the same. The wavefield at 60 μs and 80 μs acquired by the rectangle shape is smoother, because the replacement position has a small deviation from the preset. The rectangle with 3 mm length and 5 mm width is a better choice for obtaining the wavefield.

## 3. Wavefield Characteristics

The guided wave excited by pulse laser on the composite plate is dispersive and the frequency or thickness affect the propagation velocity. The wavefield characteristics of the composite with delamination can be useful for the analysis of damage factor and it can be used for quantitative assessment of damage. In this section, wavefield snapshots of the composites with single and multiple delamination defects are analyzed in space–time and frequency–wavenumber domain.

**Figure 6:**
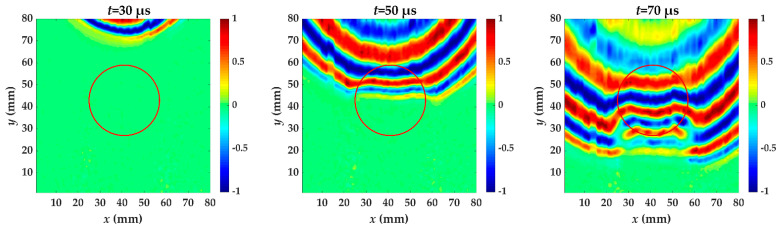
Snapshots of propagating guided wave in specimen I at different times. The red circle is the delamination made artificially in specimen I.

For specimen I, Teflon insert is between the fifth and sixth layer. Figure 6 shows the wavefield snapshots acquired from scanning area at different times. The red circle is the delamination made artificially in specimen I. In the out-of-plane displacement, the acquired wavefield shows a stronger A_0_ mode. A_0_ mode with higher amplitude has reached the delamination region at *t* = 50 μs, and the wavelength is reduced. This is because the delamination causes the incident waves to split and propagate independently through the upper and lower laminates [8], and the thickness of the measured side changes. Therefore, the wave in the delamination region propagates at a velocity lower than that in the undamaged region. Each moment subsequently is accompanied by wave pattern anomalies and a reduced wavelength.

**Figure 7:**
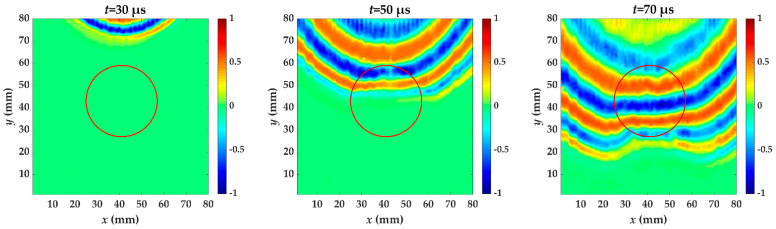
Snapshots of propagating guided wave in specimen II at different times. The red circle is the delamination made artificially in specimen II.

For specimen II, Teflon insert is between the second and third layer. Figure 7 shows snapshots of propagating guided wave at different times in specimen II. The red circle is the delamination made artificially in specimen II. The wave in the delamination region propagates at a velocity lower than that in the undamaged region. The degree of velocity slowdown in specimen II is weaker than in specimen I at *t* = 70 μs.

**Figure 8:**
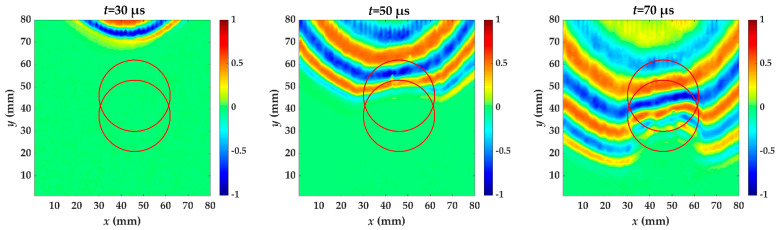
Snapshots of propagating guided wave in specimen III at different times. The red circle is the delamination made artificially in specimen III.

For specimen III, two Teflon inserts are added, one between the fifth and sixth layer, and the other second and third layer. Figure 8 shows wavefield snapshots of the propagating guided wave in specimen III at different times. The red circles are the delamination made artificially in specimen III. When the guided wave meets the delamination, a lower velocity and a reduced wavelength are also found. At *t* = 50 μs, the guided wave mainly propagates in the composite with first delamination. The propagation distance for the second crest of the guided wave is farther than specimen I and it is almost the same as specimen II. The first Teflon insert is added between the second and third layer. At *t* = 70 μs, the guided wave has passed through two delamination defects. Additionally, the wave pattern anomalies are similar to specimen I. The second Teflon insert is added between the second and third layer.

**Figure 9:**
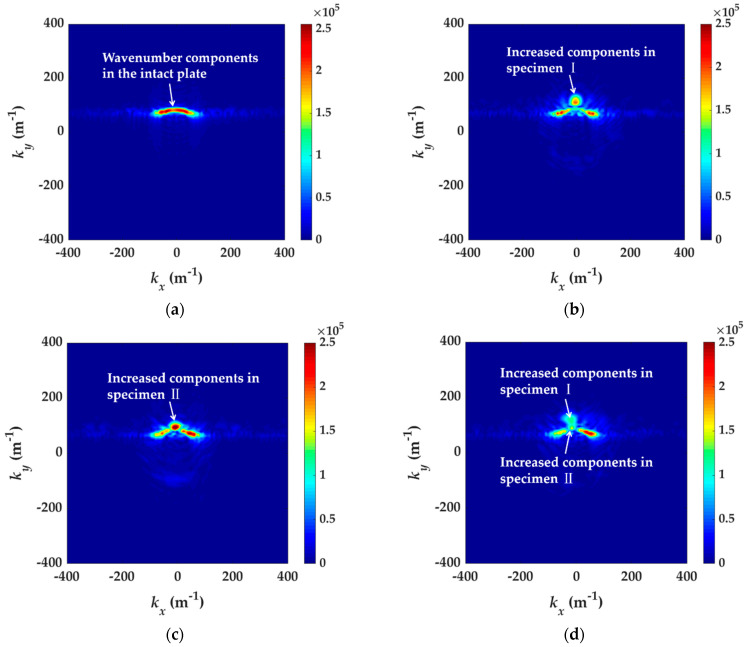
The *k_x_-k_y_* spectrums at 98 kHz. (**a**) The intact plate, (**b**) specimen I, (**c**) specimen II and (**d**) specimen III.

Frequency–wavenumber analysis methods can enable further analysis of wave modes and wavenumber distribution. By using the three-dimensional (3D) Fourier transform (FT), the acquired time–space wavefield is converted to a frequency–wavenumber field. The change in wavenumber can be extracted from the diagram of *k_x_* and *k_y_* at a specific frequency. Figure 9 shows the *k_x_-k_y_* spectrum at 98 kHz of the intact plate and three specimens. In the damaged plates, as shown in Figure 9b–d, some wavenumber components are obviously increased in addition to the original curve. The increased wavenumber component is related to delamination defects. The value of the increased wavenumber in specimen I is higher than that in specimen II. This can be explained by that the structural thickness above the delamination in specimen I is lower than that in specimen II. The increased wavenumber components appearing in specimens I and II are both found in specimen III.

The main wavenumber that exits in the intact plate, specimen I, II and III, is shown in Table 1, which is acquired from the *k_x_*-*k_y_* spectrums at 98 kHz. The wavenumber 85.94 m^−1^ is in all four specimens. The wavenumber 93.75 m^−1^ is in specimen II and III. The wavenumber 117.2 m^−1^ is in specimen I and III. It has been known in advance that the delamination defect in specimen I is preset between the fifth and sixth layer and specimen II between the second and third. From the value of increased wavenumber 93.75 m^−1^ and 117.2 m^−1^, two delamination defects exist in specimen III, one between the fifth and sixth layer, and the other between the second and third.

## 4. The Algorithm for Visualizing Defects

**Figure 10:**
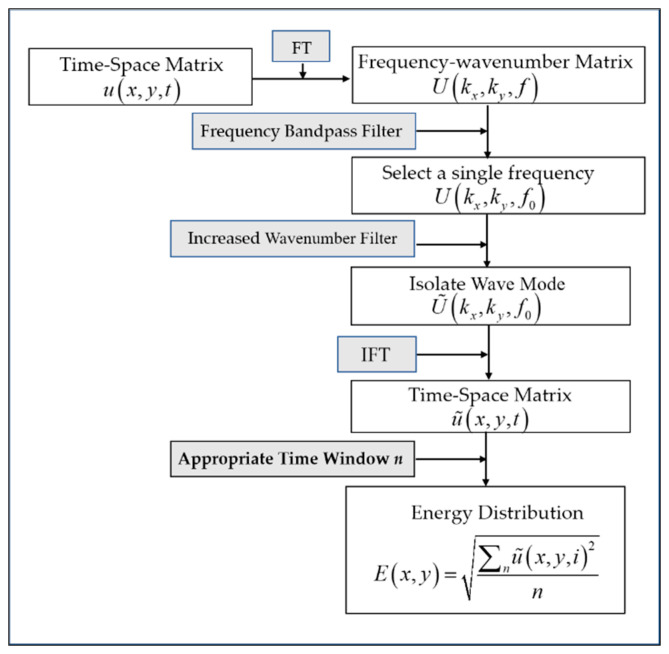
Algorithm of localized wave energy with appropriate time windows for delamination detection.

Taking the time window for standard ultrasonic testing as a reference, the algorithms of localized wave energy with appropriate time windows are present. The algorithm gathers wave energy anomalies caused by delamination defects at a certain frequency. Figure 10 shows the algorithms of localized wave energy with appropriate time windows.

First, experimental equipment is used to acquire the 3D measurement matrix. Then, the signal processing steps below are followed.

(1) The 3D full-wavefield matrix u(x,y,t) is transformed from the space–time domain to the frequency–wavenumber domain using the 3D Fourier transform and the mathematical formula is expressed as Equation (1). To obtain the wavenumber field information at frequency $f_{0}$, a narrow frequency bandpass filter $W_{F}$ is applied, shown as Equation (2).

(1) $$U\left(k_{x},k_{y},f\right)=F_{3D}\left[u\left(x,y,t\right)\right]$$

(2) $$U\left(k_{x},k_{y},f_{0}\right)=U\left(k_{x},k_{y},f\right)W_{F}$$

(2) Wavenumber filter $W_{k}\left(k_{x},k_{y},f_{0}\right)$ is applied in the wavenumber domain to extract the increased wavenumber, described as Equation (3). Additionally, the wavenumber filter $W_{k}\left(k_{x},k_{y},f_{0}\right)$ is given in Equation (4). The *Threshold* is decided by the wavenumber of A_0_ mode at frequency $f_{0}$ in the intact plate. The frequency $f_{0}$ used in this study is 98 kHz. Other frequencies can also be selected and the effect of the frequency on the quality of the results has not been studied.

(3) $$\tilde{U}\left(k_{x},k_{y},f_{0}\right)=U\left(k_{x},k_{y},f_{0}\right)W_{k}\left(k_{x},k_{y},f_{0}\right)$$

(4) $$W_{k}\left(k_{x},k_{y},f_{0}\right)=\{\begin{array}{c} 0\;\;\;\;\;\;\;\left|k\right|<Threshold\\ 1\;\;\;\;\;\;\;\left|k\right|<Threshold\; \end{array}$$

(3) An inverse Fourier transform is then applied, so that the wavefield with increased wavenumber components can be obtained by Equation (5). By observing the energy diagrams at different times, the appropriate time windows n can be selected to highlight the energy anomalies and i represents the selected moment. The energy anomalies can be called localized wave energy. The energy anomalies distribution can be calculated by Equation (6), and delamination defects can be identified by localized wave energy.

(5) $$\tilde{u}\left(x,y,t\right)=F_{3D}^{-1}\left[\tilde{U}\left(k_{x},k_{y},f_{0}\right)\right]$$

(6) $$E\left(x,y\right)=\sqrt{\frac{\sum_{n}\tilde{u}{\left(x,y,i\right)}^{2}}{n}}$$

## 5. Experimental Results

### 5.1. Single Defect

**Figure 11:**
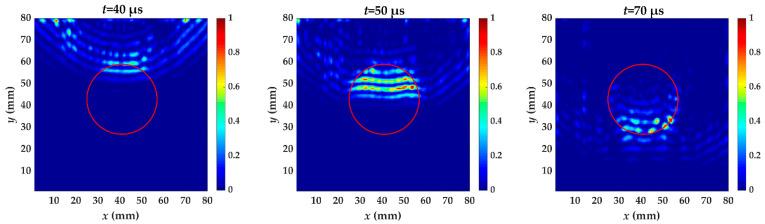
The energy distribution of specimen I caused by the increased wavenumber at *t* = 40, 50 and 70 μs. The red circle is the delamination made artificially in specimen I.

**Figure 12:**
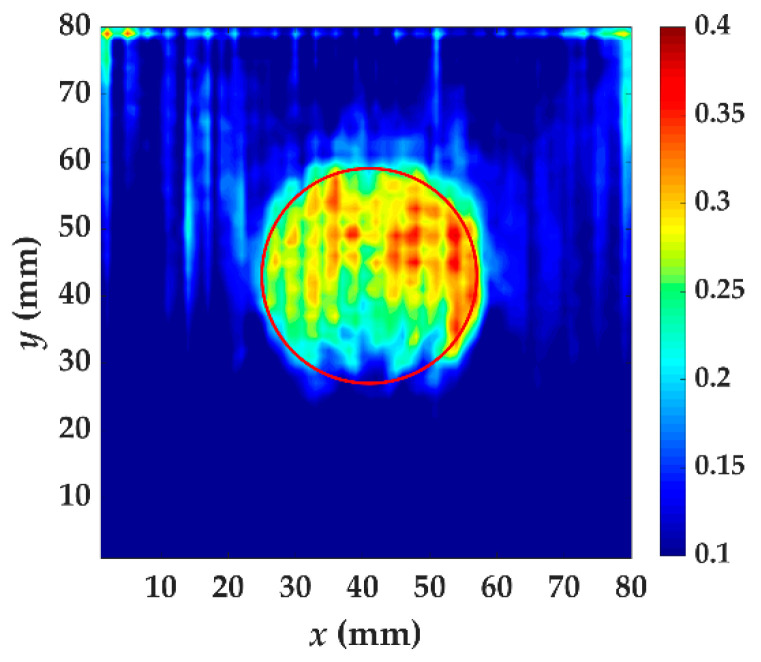
The test result of specimen I using localized wave energy algorithm. The time windows are from *t* = 40 μs to *t* = 70 μs. The red circle is the delamination made artificially in specimen I.

In specimen I, the energy caused by increased wavenumber component is becoming larger when passing through the delamination, as shown in Figure 11. This is because the thickness of guided wave penetration on the measured side is reduced. The delamination causes the whole specimen to be divided into two parts and the thickness of the two parts is reduced. What we are concerned about is the thickness change of the measured side. The guided wave excited by a laser is dispersive and the thickness affects the propagation at a certain frequency. When the guided wave has completely passed the delamination, there still exits trapping wave energy. The energy anomalies can be used for locating where delamination damage occurs. From *t* = 40 μs to *t* = 70 μs, the phenomenon of energy anomalies is obvious, indicating that these are the appropriate time windows. The test result of specimen I using localized wave energy algorithm is shown in Figure 12. A clear profile of delamination defect can be obtained by this algorithm. The value of increased wavenumber (117.2 m^−1^) indicates that the Teflon insert is between the fifth and sixth layer.

**Figure 13:**
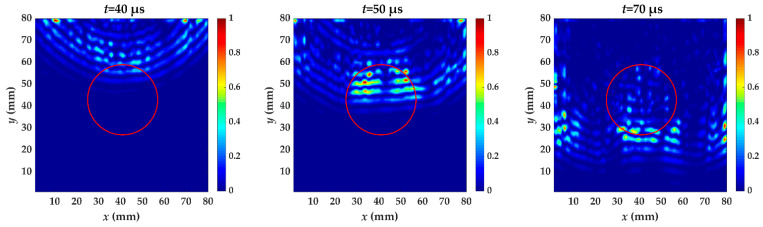
The energy distribution of specimen II caused by the increased wavenumber at *t* = 40, 50 and 70 μs. The red circle is the delamination made artificially in specimen II.

**Figure 14:**
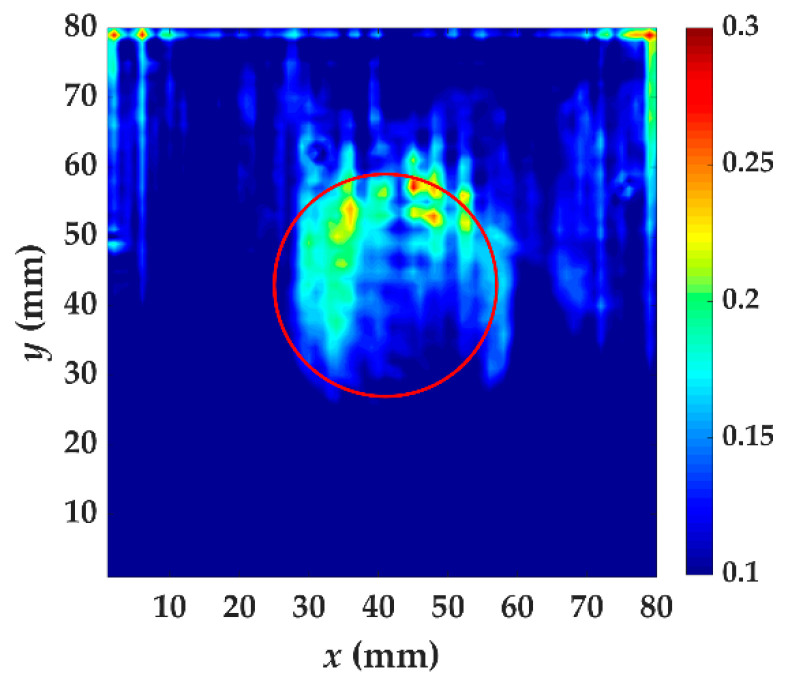
The test result of specimen II using localized wave energy algorithm. The time windows are from *t* = 40 μs to *t* = 70 μs. The red circle is the delamination made artificially in specimen II.

Similar to the phenomenon of specimen I, the energy caused by increased wavenumber component in specimen II is also becoming larger when passing through the delamination and it is less obvious, as shown in Figure 13. The time windows from *t* = 40 μs to *t* = 70 μs can also be used for specimen II. The test result of specimen II using the localized wave energy algorithm is shown in Figure 14. The delamination profile is visually close to the actual one. The value of increased wavenumber (93.75 m^−1^) indicates that the Teflon insert is between the second and third layer. The result can show a successful identification of delamination location, size and depth.

### 5.2. Multiple Defects

**Figure 15:**
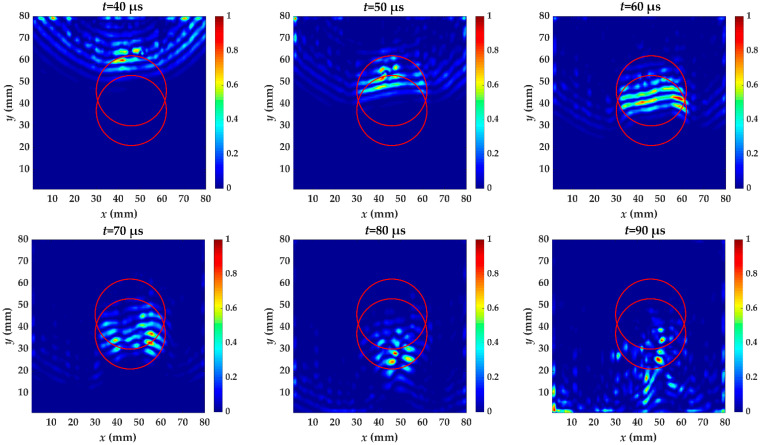
The energy distribution of specimen III caused by the increased wavenumber at *t* = 40~90 μs with 10 μs intervals. The red circles are the delamination made artificially in specimen III.

**Figure 16:**
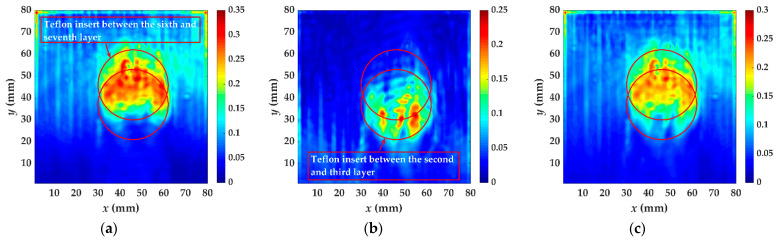
The test result of specimen III using localized wave energy algorithm: (**a**) the time windows are from *t* = 40 μs to *t* = 60 μs, (**b**) the time windows are from *t* = 60 μs to *t* = 90 μs, (**c**) the time windows are from *t* = 40 μs to *t* = 90 μs. The red circles are the delamination made artificially in specimen III.

For specimen III, there are two artificial delamination defects, one located between the fifth and sixth layer, and the other between the second and third. The energy distribution caused by the increased wavenumber at *t* = 40~90 μs with 10 μs intervals is shown in Figure 15. When the guided waves pass through the first delamination, the energy caused by the increased wavenumber component is becomes large in the first delamination region from *t* = 40 μs to *t* = 60 μs. The energy discontinuity of the defect area at 70 μs is obviously different from previous time and the contour of the energy accumulation has changed, indicating that the guided wave has spread to the second defect. When the wavefield caused by the increased wavenumber has passed through the second defect, shown at 90 μs, some energy still remains inside the delamination. The second time windows are from *t* = 60 μs to *t* = 90 μs. Figure 15 shows the localized wave energy distribution when the time windows are from *t* = 40 μs to 90 μs, and the result is not affected by the choice of time windows. The defect overlap region shows high energy anomalies. The imaging results of two defects are shown in Figure 16a, b, respectively. The appropriate time window is meaningful for identifying each delamination defect.

Combined with the wavefield characteristics in Section 3 and the value of the increased wavenumber components (93.75 and 117.2 m^−1^ at Table 1), the first delamination is between the second and third layer, and the second is between the fifth and sixth. Different from the methods in the previous studies, two delamination profiles can be obtained, respectively, by the algorithm. If the upper (laser measurement side) delamination fully covers the lower one, the localized wave energy of the overlapping area is high. The region of high localized wave energy is the lower delamination defect. If the boundaries of the delamination defects coincide, the number of delamination defects and coincidence degree should be determined by increased wavenumber value and the localized wave energy.

## 6. Conclusions

In this paper, aluminum foil was pasted onto the surface of the composite because of its low thermal conductivity. Using the fully noncontact system of fixed-point laser excitation and scanning laser sensor, the circular aluminum foil with a 6 mm diameter and the rectangular one with 3 mm length and 5 mm width were tested in the experiment to study the effects of their sizes and shapes on the wavefield. By comparing the wavefield snapshots obtained by two aluminum foils, the rectangle with 3 mm length and 5 mm width is a better choice because the replacement position has a small deviation from the preset.

Taking the time window for standard ultrasonic testing as a reference, the algorithms of localized wave energy with appropriate time windows are present for the detection of single and multiple delamination defects. The composites with multiple delamination defects were first researched using the method of localized wave energy. Since the material properties of CFRP are not known in advance, two specimens with single delamination defects were used to study the wavenumber change related to material properties. For two specimens with different layered Teflon inserts, the propagation characteristics of the guided wave is different. For specimen I where Teflon is inserted between the fifth and sixth layer, the wavefield distortion is more obvious and the value of increased wavenumber is higher than in specimen II where Teflon is inserted between the second and third layer. Energy distribution caused by the increased wavenumber is becoming large when passing through the delamination. For specimen III, where two Teflon inserts are between the fifth/second and sixth/third layer, the frequency–wavenumber curve shows two increased wavenumber components appearing in two composites with a single delamination defect. The algorithms of localized wave energy with appropriate time windows are not only suitable for the detection of single delamination defect, but also for multiple delamination defects. For the detection of multiple delamination defects, the total localized wave energy distribution is not affected by the choice of time windows and the defect overlap region shows high energy anomalies. The appropriate time windows are meaningful for understanding the distribution of each delamination defect. They show a successful identification of the delamination location and size. Combined with the analysis of the wavefield and the value of the increased wavenumber, the position in the thickness direction can be determined.

A fully noncontact inspection method for the composites was present through non-ablative way. The limitation of the method of pasting aluminum foil onto composite material is that aluminum foil would be ablated after a period of time and it needs to be replaced by another material. The algorithms of localized wave energy with appropriate time windows have only been verified in composites with two regular-shaped delamination defects, and it should be verified in irregular-shaped delamination defects. In the future, we will focus on the influence of different materials on the ultrasonic wavefield of CFRP as well as the detection of multiple and irregular delamination defects.

## Figures and Tables

**Table 1:** Main wavenumber that exits in the intact plate, specimen I, II and III.

Main Wavenumber in the Intact Plate (m^−1^)	Main Wavenumber in the Specimen Ⅰ (m^−1^)	Main Wavenumber in the Specimen Ⅱ (m^−1^)	Main Wavenumber in the Specimen Ⅲ (m^−1^)
85.94	85.94	85.94	85.94
		93.75	93.75
	117.2		117.2

## Data Availability

The data presented in this study are available on reasonable request from the corresponding author.

## References

[1] Qing X., Li W., Wang Y., Sun H. (2019). Piezoelectric Transducer-Based Structural Health Monitoring for Aircraft Applications. Sensors.

[2] Li B., Liu Y., Gong K., Li Z. (2013). Damage localization in composite laminates based on a quantitative expression of anisotropic wavefront. Smart Mater. Struct..

[3] Kazys R., Sliteris R., Mazeika L., Tumsys O., Zukauskas E. (2019). Attenuation of a Slow Subsonic A0 Mode Ultrasonic Guided Wave in Thin Plastic Films. Materials.

[4] Ruzzene M. (2007). Frequency-wavenumber domain filtering for improved damage visualization. Smart Mater. Struct..

[5] Ling E.H., Abdul Rahim R.H. (2020). A review on ultrasonic guided wave technology. Aust. J. Mech. Eng..

[6] Green R.E. (2004). Non-contact ultrasonic techniques. Ultrasonics.

[7] Ostachowicz W., Radzienski M., Kudela P. (2014). 50th Anniversary Article: Comparison Studies of Full Wavefield Signal Processing for Crack Detection. Strain.

[8] Sohn H., Dutta D., Yang J.Y., DeSimio M., Olson S., Swenson E. (2011). Automated detection of delamination and disbond from wavefield images obtained using a scanning laser vibrometer. Smart Mater. Struct..

[9] Hong S.C., Abetew A.D., Lee J.R., Ihn J.B. (2017). Three dimensional evaluation of aluminum plates with wall-thinning by full-field pulse-echo laser ultrasound. Opt. Lasers Eng..

[10] Yu L., Tian Z. (2013). Lamb wave Structural Health Monitoring Using a Hybrid PZT-Laser Vibrometer Approach. Struct. Heal. Monit..

[11] Li Z., He C., Liu Z., Wu B. (2019). Quantitative detection of lamination defect in thin-walled metallic pipe by using circumferential Lamb waves based on wavenumber analysis method. NDT E Int..

[12] Dubois M., Drake T.E. (2011). Evolution of industrial laser-ultrasonic systems for the inspection of composites. Nondestruct. Test. Eval..

[13] Kusano M., Hatano H., Watanabe M., Takekawa S., Yamawaki H., Oguchi K., Enoki M. (2018). Mid-infrared pulsed laser ultrasonic testing for carbon fiber reinforced plastics. Ultrasonics.

[14] Derusova D., Vavilov V., Sfarra S., Sarasini F., Krasnoveikin V., Chulkov A., Pawar S. (2019). Ultrasonic spectroscopic analysis of impact damage in composites by using laser vibrometry. Compos. Struct..

[15] Juarez P.D., Leckey C.A.C. (2015). Multi-frequency local wavenumber analysis and ply correlation of delamination damage. Ultrasonics.

[16] Chia C.C., Lee J.-R., Park C.-Y., Jeong H.-M. (2012). Laser ultrasonic anomalous wave propagation imaging method with adjacent wave subtraction: Application to actual damages in composite wing. Opt. Laser Technol..

[17] Flynn E.B., Chong S.Y., Jarmer G.J., Lee J.-R. (2013). Structural imaging through local wavenumber estimation of guided waves. NDT E Int..

[18] An Y., Park B., Sohn H. (2013). Complete noncontact laser ultrasonic imaging for automated crack visualization in a plate. Smart Mater. Struct..

[19] Zhang K., Zhou Z. (2018). Quantitative characterization of disbonds in multilayered bonded composites using laser ultrasonic guided waves. NDT E Int..

[20] Gao T., Sun H., Hong Y., Qing X. (2020). Hidden Corrosion Detection Using Laser Ultrasonic Guided Waves with Multi-frequency Local Wavenumber Estimation. Ultrasonics.

[21] Yu X., Ratassepp M., Fan Z. (2017). Damage detection in quasi-isotropic composite bends using ultrasonic feature guided waves. Compos. Sci. Technol..

[22] Zhao G., Wang B., Wang T., Hao W., Luo Y. (2019). Detection and monitoring of delamination in composite laminates using ultrasonic guided wave. Compos. Struct..

[23] Xu C., Yang Z., Zhai Z., Qiao B., Tian S., Chen X. (2019). A weighted sparse reconstruction-based ultrasonic guided wave anomaly imaging method for composite laminates. Compos. Struct..

[24] Kim Y.-H., Kim D.-H., Han J.-H., Kim C.-G. (2007). Damage assessment in layered composites using spectral analysis and Lamb wave, Compos. Compos. Part B Eng..

[25] Choi Y., Lee J.-R. (2017). Multi-directional adjacent wave subtraction and shifted time point mapping algorithms and their application to defect visualization in a space tank liner. NDT E Int..

[26] Lee J.R., Ciang Chia C., Jin Shin H., Park C.Y., Jin Yoon D. (2011). Laser ultrasonic propagation imaging method in the frequency domain based on wavelet transformation. Opt. Lasers Eng..

[27] Michaels T.E., Michaels J.E., Ruzzene M. (2011). Frequency–wavenumber domain analysis of guided wavefields. Ultrasonics.

[28] Park B., An Y., Sohn H. (2014). Visualization of hidden delamination and debonding in composites through noncontact laser ultrasonic scanning. Compos. Sci. Technol..

[29] Tian Z., Yu L., Leckey C., Seebo J. (2015). Guided wave imaging for detection and evaluation of impact-induced delamination in composites. Smart Mater. Struct..

[30] Rogge M.D., Leckey C.A.C. (2013). Characterization of impact damage in composite laminates using guided wavefield imaging and local wavenumber domain analysis. Ultrasonics.

[31] Kudela P., Radzienski M., Ostachowicz W. (2018). Impact induced damage assessment by means of Lamb wave image processing. Mech. Syst. Signal Process..

[32] Chang H.-Y., Yuan F.-G. (2019). Damage imaging in a stiffened curved composite sandwich panel with wavenumber index via Riesz transform. Struct. Health Monit..

[33] Leckey C.A.C., Seebo J.P. (2015). Guided wave energy trapping to detect hidden multilayer delamination damage. AIP Conf. Proc..

[34] Mei H., Giurgiutiu V. (2020). Characterization of multilayer delaminations in composites using wavenumber analysis: Numerical and experimental studies. Struct. Health Monit..

[35] Laureti S., Ricci M., Mohamed M.N.I.B., Senni L., Davis L.A.J., Hutchins D.A. (2018). Detection of rebars in concrete using advanced ultrasonic pulse compression techniques. Ultrasonics.

[36] Abetew A.D., Truong T.C., Hong S.C., Lee J.R., Ihn J.B. (2020). Parametric optimization of pulse-echo laser ultrasonic system for inspection of thick polymer matrix composites. Struct. Health Monit..

